# Survival after Locoregional Recurrence or Second Primary Breast Cancer: Impact of the Disease-Free Interval

**DOI:** 10.1371/journal.pone.0120832

**Published:** 2015-04-10

**Authors:** Annemieke Witteveen, Annemiek B. G. Kwast, Gabe S. Sonke, Maarten J. IJzerman, Sabine Siesling

**Affiliations:** 1 Department of Health Technology and Services Research, MIRA Institute of Biomedical Technology and Technical Medicine, Twente University, Enschede, The Netherlands; 2 Department of Registration and Research, Comprehensive Cancer Centre the Netherlands (IKNL), Utrecht, the Netherlands; 3 Department of Medical Oncology, Netherlands Cancer Institute, Antoni van Leeuwenhoek Hospital, Amsterdam, the Netherlands; INRS, CANADA

## Abstract

The association between the disease-free interval (DFI) and survival after a locoregional recurrence (LRR) or second primary (SP) breast cancer remains uncertain. The objective of this study is to clarify this association to obtain more information on expected prognosis. Women first diagnosed with early breast cancer between 2003–2006 were selected from the Netherlands Cancer Registry. LRRs and SP tumours within five years of first diagnosis were examined. The five-year period was subsequently divided into three equal intervals. Prognostic significance of the DFI on survival after a LRR or SP tumour was determined using Kaplan-Meier estimates and multivariable Cox regression analysis. Follow-up was complete until January 1, 2014. A total of 37,278 women was included in the analysis. LRRs or SP tumours were diagnosed in 890 (2,4%) and 897 (2,4%) respectively. Longer DFI was strongly and independently related to an improved survival after a LRR (long versus short: HR 0.65, 95% CI 0.48–0.88; medium versus short HR 0.81, 95% CI 0.65–1.01). Other factors related to improved survival after LRR were younger age (<70 years) and surgical removal of the recurrence. No significant association was found between DFI and survival after SP tumours. This is the first study to explore the association between the DFI and survival after recurrence in a nationwide population-based cancer registry. The DFI before a LRR is an independent prognostic factor for survival, with a longer DFI predicting better prognosis.

## Introduction

Breast cancer-related mortality is decreasing in many countries because of earlier diagnosis and improved treatment modalities [1,2]. After treatment patients receive follow-up care to improve life expectancy by detecting recurrence of breast cancer in an asymptomatic stage. Recurrence of breast cancer can be a local recurrence (LR), regional recurrence (RR), second primary (SP) tumour or distant metastasis (DM). Early detection and treatment of DM does not lead to better outcomes and is therefore not an aim of follow-up care. Presence of a LR and/or RR can be defined as locoregional recurrence (LRR). The majority of LRRs are diagnosed within five years of the primary tumour [3]. LRR rates vary between 3% for patients with stage T1N0 who underwent mastectomy to 13% for patients with nodal involvement and breast-conserving surgery [4–6]. SP breast cancer (diagnosed at least three months from the primary tumour diagnosis [7] and with different primary location and morphology) occurs in 2–6% of the patients [8–12].

The time between treatment of the primary tumour and the detection of a recurrence is known as the disease-free interval (DFI). The effect of the DFI on the prognosis after a LRR or SP tumour is not well established. Previous reports defined short DFI as the first five years after the primary tumour and long as the following years [13–16], or results were based on a single institution or small patient series [17–20]. If the distinction is made of a short DFI being within the first five years after diagnosis and a long DFI the following years, a long DFI is associated with favourable outcomes [13–16]. In a study with 391 women treated with breast-conserving surgery and radiotherapy Fredriksson et al. [21] also observed that a short DFI before a LR has a negative impact on survival. Yet based on 133 patients from two randomised trials Van Tienhoven et al. [3] found the DFI to be prognostic only for the time to subsequent recurrence and not an independent prognostic factor for survival. The aim of this study was to explore the relation between the DFI and survival after a LRR or SP tumour within five years of treatment of the primary breast cancer, based on a nationwide population.

## Patients and Methods

### Study population

Patients were selected from the Netherlands Cancer Registry (NCR), a nationwide population-based registry, which has recorded almost all newly diagnosed tumours since 1989. Notifications are obtained from the nationwide Pathology Automated Archives (PALGA). Specially trained registration clerks record the information on patient, tumour and treatment characteristics directly from the patient files, as well as data concerning recurrences within the first five years following primary breast cancer. The information from the patient records was anonymized and de-identified prior to analysis by the Comprehensive Cancer Centre the Netherlands (IKNL). The authors had consent from the Advisory Committee of the IKNL. The study did not need approval of the Medical Ethical Committee (METc), while there was no direct patient contact and according to local regulations in the Netherlands (WMO) only studies with high burden for patients have to be reviewed.

Women diagnosed with primary invasive breast cancer between 2003 and 2006 without DM, previous, or synchronous tumours (diagnosed within three months after the first tumour [7]), treated with curative intent and without neo-adjuvant systemic treatment were selected from the registry (N = 37,278). Patients were treated with curative intent if the tumour was surgically removed and no macroscopic residual disease was left. In case of microscopic residue, the patient should have had adjuvant treatment. Of the selected patients, 1,787 (4.8%) developed a LRR or SP tumour within the first five years following primary breast cancer treatment. Only first or synchronous LRRs or SP tumours were considered.

### Statistical analyses

The DFI was measured from the date of final surgery of the primary tumour to the date of LRR or SP tumour diagnosis. To analyse the effect of the DFI on survival after a LRR or SP tumour, the five year period after the primary breast cancer was divided into three equal intervals: (1) patients with a LRR or SP tumour diagnosed within 608 days were considered to have a short DFI; (2) between 609–1217 days a medium DFI; and (3) after 1217 days a long DFI. Overall survival time was measured from the date of final surgery of the primary tumour to death or the end of the study period (January 1, 2014) and was used for the comparison with women without a recurrence. Survival after the LRR or SP tumour was used for the comparisons between the different DFI categories and determined from the date of diagnosis of the LRR or SP tumour until death or the end of the study period. Follow-up was complete by linkage with the Dutch municipal civil registry until January 1, 2014, after which surviving patients were censored.

The patient, tumour, and treatment characteristics shown in Tables 1 and 2 were assessed for their influence on survival after a LRR or SP tumour using Kaplan-Meier estimates and the log-rank test for equality of survivor functions. To reveal differences in the characteristics between categorical variables, χ^2^ tests were used. Variables with probability values <0.1 on the log-rank test were included in the multivariable analysis. In the Cox proportional hazards model backward selection was used to find the variables with significant influence on survival after the LRR or SP tumour; p-values of <0.05 were considered statistically significant. This led to inclusion of the following variables: DFI category, type of recurrence, age, tumour grade according to Bloom and Richardson [22], size, lymph node status and hormone receptor status (ER and PR) of the primary tumour, and surgical removal of the recurrence. Interaction between the variables was tested by adding interaction terms into the model. The Cox proportional hazards assumption was tested both numerically and graphically. Stratification into short, medium and long DFIs and stratification for LRRs and SP tumours was performed to reveal possible differences in the influence of the covariates for the different categories.

**Table 1:** Patient and primary tumour characteristics, for LRRs stratified by length of the DFI and tested for differences between the DFI groups.

		Without LRR/SP	SP		LRR		Length of DFI						P
							*Short*		*Medium*		*Long*		
		%	N	%	N	%	N	%	N	%	N	%	
**Total**		35,491	897		890		299		348		243		
**Age category**													0.034
	<70	77.3	713	79.5	690	77.5	221	73.9	267	76.7	202	83.1	
	≥70	22.7	184	20.5	200	22.5	78	26.1	81	23.3	41	16.9	
**Histologic type**													0.048
	Ductal	79.4	672	74.9	740	83.1	263	88.0	288	82.8	189	77.8	
	Lobular	10.7	107	11.9	82	9.2	20	6.7	29	8.3	33	13.6	
	Mixed	4.2	54	6.0	27	3.0	5	1.7	13	3.7	9	3.7	
	Other	5.7	64	7.1	41	4.6	11	3.7	18	5.2	12	4.9	
**Grade**													<0.001
	I,II	66.9	644	76.6	440	54.0	115	41.8	167	52.5	158	71.2	
	III	33.1	197	23.4	375	46.0	160	58.2	151	47.5	64	28.8	
	Unknown		56		75		24		30		21		
**Tumour size**													<0.001
	≤2 cm	61.1	643	72.3	469	53.7	121	41.4	194	56.4	154	64.7	
	>2 cm	38.8	246	27.7	405	46.3	171	58.6	150	43.6	84	35.3	
	Unknown		8		16		7		4		5		
**Multifocal**													0.238
	No	84.8	558	81.8	507	79.1	184	80.7	187	75.7	136	81.9	
	Yes	15.2	124	18.2	134	20.9	44	19.3	60	24.3	30	18.1	
	Unknown		215		249		71		101		77		
**Lymph node status**													0.025
	Negative	61.3	660	74.7	503	58.1	155	53.1	189	56.3	159	66.8	
	1–3 positive	27.5	166	18.8	244	28.2	90	30.8	100	29.8	54	22.7	
	>3 positive	11.2	58	6.6	119	13.7	47	16.1	47	14.0	25	10.5	
	Unknown		13		24		7		12		5		
**ER status**													<0.001
	Negative	18.8	121	17.3	219	31.9	116	48.9	75	28.3	28	15.1	
	Positive	81.2	578	82.7	468	68.1	121	51.1	190	71.7	157	84.9	
	Unknown		198		203		62		83		58		
**PR status**													<0.001
	Negative	33.7	223	31.9	330	48.7	156	66.7	120	46.2	54	29.5	
	Positive	66.3	475	68.1	347	51.3	78	33.3	140	53.8	129	70.5	
	Unknown		199		213		65		88		60		
**Her2-Neu status**													0.347
	Negative	85.2	346	88.7	278	82.7	92	81.4	113	80.7	73	88.0	
	Positive	14.8	44	11.3	58	17.3	21	18.6	27	19.3	10	12.0	
	Unknown		507		554		186		208		160		

Abbreviations: LRR = locoregional recurrence, SP = second primary, DFI = disease-free interval, ER = oestrogen receptor, PR = progesterone receptor, Her2-Neu = human epidermal growth factor receptor 2

**Table 2:** Primary treatment and recurrence characteristics, for LRRs stratified by length of the DFI and tested for differences between the DFI groups.

			Without LRR/SP	SP		LRR		Length of DFI						P
								*Short*		*Medium*		*Long*		
			%	N	%	N	%	N	%	N	%	N	%	
Number of surgeries														0.684
	1		88.9	766	85.4	780	87.6	259	86.6	303	87.1	218	89.7	
	2		10.5	124	13.8	100	11.2	36	12.0	40	11.5	24	9.9	
	≥3		0.6	7	0.8	10	1.1	4	1.3	5	1.4	1	0.4	
Type of surgery														<0.001
	Breast conserving		56.3	517	57.6	395	44.4	95	31.8	156	44.8	144	59.3	
	Non-breast conserving		43.5	380	42.4	495	55.6	204	68.2	192	55.2	99	40.7	
Time from incidence to last surgery														0.145
	<30 days		74.0	619	69.0	637	71.6	207	69.2	250	71.8	180	74.1	
	30–60 days		22.0	223	24.9	207	23.3	69	23.1	82	23.6	56	23.0	
	>60 days		4.0	55	6.1	46	5.2	23	7.7	16	4.6	7	2.9	
Axillary lymph node dissection														0.004
	No		49.4	545	60.8	425	47.8	126	42.1	162	46.6	137	56.4	
	Yes		50.7	352	39.2	465	52.2	173	57.9	186	53.4	106	43.6	
Chemotherapy														0.003
	No		64.1	692	77.1	579	65.1	177	59.2	224	64.4	178	73.3	
	Yes		35.9	205	22.9	311	34.9	122	40.8	124	35.6	65	26.7	
Radiotherapy														<0.001
	No		34.3	313	34.9	447	50.2	184	61.5	174	50.0	89	36.6	
	Yes		65.7	584	65.1	443	49.8	115	38.5	174	50.0	154	63.4	
Hormone therapy														0.054
	No		58.2	682	76.0	643	72.2	231	77.3	245	70.4	167	68.7	
	Yes		41.8	215	24.0	247	27.8	68	22.7	103	29.6	76	31.3	
Length of DFI														
	Short		na	248	27.6	na		na		na		na		
	Medium			334	37.2									
	Long			315	35.1									
Type of recurrence														0.378
	LR		na	na		584	65.6	184	61.5	233	67.0	167	68.7	
	RR					240	27.0	88	29.4	93	26.7	59	24.3	
	Synchronous LRRs					66	7.4	27	9.0	22	6.3	17	7.0	
Surgery of LRR/SP														0.001
	No		na	38	4.2	368	41.3	147	49.2	138	39.7	83	34.2	
	Yes			859	95.8	522	58.7	152	50.8	210	60.3	160	65.8	
Chemotherapy of LRR/SP														0.172
	No		na	705	78.6	625	70.2	198	66.2	250	71.8	177	72.8	
	Yes			192	21.4	265	29.8	101	33.8	98	28.2	66	27.2	
Radiotherapy of LRR/SP														<0.001
	No		na	467	52.1	432	48.5	118	39.5	172	49.4	142	58.4	
	Yes			430	47.9	458	51.5	181	60.5	176	50.6	101	41.6	
Hormone therapy of LRR/SP														0.001
	No		na	597	66.6	562	63.1	213	71.2	211	60.6	138	56.8	
	Yes			300	33.4	328	36.9	86	28.8	137	39.4	105	43.2	

Abbreviations: LRR = locoregional recurrence, SP = second primary, LR = local recurrence, RR = regional recurrence, na = not applicable.

Missing values from the variables included in the Cox proportional hazards model were multiple imputed using chained equations [23, 24]. The HER2neu status had a relatively high percentage of missing values (60%) while it has only been registered on a nation-wide basis since 2004. Missing values were considered to occur randomly, which validates the use of imputation. All analyses were performed using STATA version 12 software.

## Results

### Characteristics

Of the 1,787 patients with a recurrence, 33% developed a LR, 13% a RR, 4% synchronous LRRs and 50% SP breast cancer. Almost all of the SP tumours (95%) were contralateral. Median length of the DFI after treatment of the primary tumour was 2.5 years. Compared to women with LRRs, women with SP tumours had less often ductal primary tumours, had a smaller primary tumour with a lower grade, less nodal involvement and more often a positive hormone status (both ER and PR).

### Treatment

The treatment of the LRR appeared to diverge from the treatment of the primary tumour (Table 3). Of the women with a LRR, 59% received surgery; 76% of the women with a LR and 15% with a RR. The patients without surgery for their LRR were treated with radiotherapy in 59% of the cases. In contrast, the treatment of SP tumours only diverged from the treatment of the primary tumour with regard to the same type of therapy (Table 4). The SP tumours were surgically removed in 96% of the cases. Differences in treatment of LRRs were also found when comparing the DFI groups: women with a LRR and a short DFI received less surgery, more radiotherapy and less hormone therapy compared to women with a medium or long DFI (Table 2).

**Table 3:** Treatment of the primary tumour against the treatment of LRRs.

		Primary tumour							
		*Breast-conserving surgery*		*Chemotherapy*		*Radiotherapy*		*Hormone therapy*	
LRR		No	Yes	No	Yes	No	Yes	No	Yes
		(n = 506)	(n = 395)	(n = 589)	(n = 312)	(n = 447)	(n = 454)	(n = 649)	(n = 252)
*Surgery*	No	29%*	13%*	24%*	18%*	24%*	18%*	28%*	13%*
	(n = 385)								
	Yes	27%*	32%*	41%*	17%*	26%*	32%*	44%*	15%*
	(n = 516)								
*Chemotherapy*	No	40%	30%	51%*	19%*	38%*	32%*	49%*	21%*
	(n = 682)								
	Yes	16%	14%	14%*	16%*	12%*	17%*	23%*	7%*
	(n = 219)								
*Radiotherapy*	No	19%*	30%*	33%*	15%*	17%*	32%*	37%	12%
	(n = 457)								
	Yes	37%*	15%*	32%*	20%*	33%*	18%*	36%	16%
	(n = 444)								
*Hormone therapy*	No	34%	29%	36%*	27%*	29%*	34%*	49%*	14%*
	(n = 589)								
	Yes	22%	15%	29%*	8%*	21%*	16%*	23%*	14%*
	(n = 312)								

* indicates a significant difference Abbreviations: LRR = locoregional recurrence.

**Table 4:** Treatment of the primary tumour against the treatment of SP tumours.

		Primary tumour							
		*Breast-conserving surgery*		*Chemotherapy*		*Radiotherapy*		*Hormone therapy*	
SP		No	Yes	No	Yes	No	Yes	No	Yes
		(n = 299)	(n = 446)	(n = 585)	(n = 160)	(n = 248)	(n = 497)	(n = 585)	(n = 160)
*Surgery*	No	3%*	1%*	4%	1%	3%*	2%*	3%	1%
	(n = 34)								
	Yes	39%*	56%*	73%	22%	32%*	63%*	73%	23%
	(n = 711)								
*Chemotherapy*	No	34%	44%	64%*	14%*	29%	50%	60%*	19%*
	(n = 551)								
	Yes	8%	13%	13%*	9%*	6%	15%	16%*	5%*
	(n = 194)								
*Radiotherapy*	No	33%*	19%*	39%	13%	28%*	25%*	37%*	15%*
	(n = 373)								
	Yes	9%*	38%*	38%	10%	7%*	41%*	39%*	9%*
	(n = 372)								
*Hormone therapy*	No	27%	39%	51%	15%	22%	45%	53%*	14%*
	(n = 477)								
	Yes	15%	18%	26%	7%	13%	20%	24%*	10%*
	(n = 268)								

* indicates a significant difference. Abbreviations: SP = second primary.

### Survival

**Fig 1:**
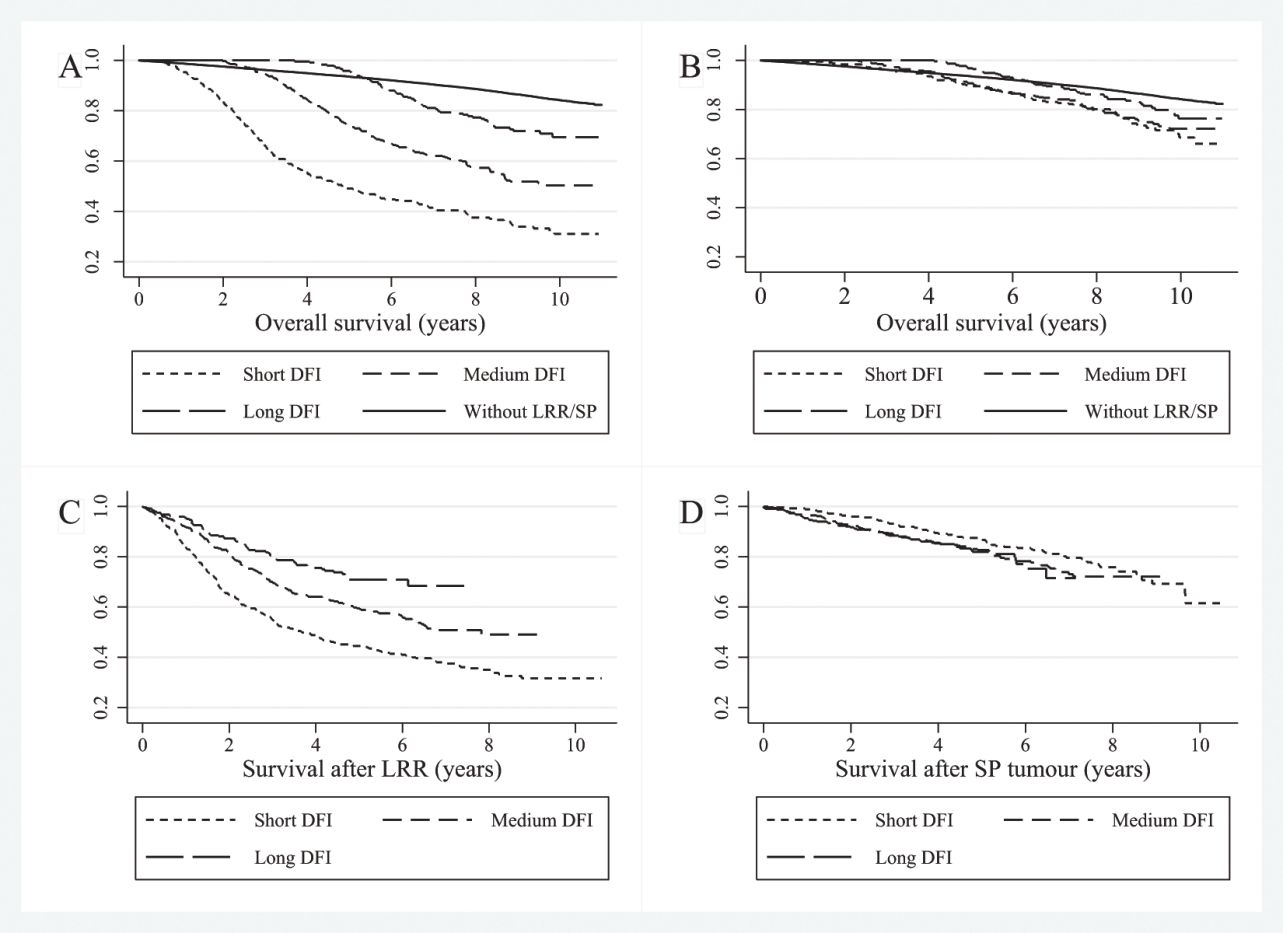
Kaplan-Meier curves divided in short, medium and long DFIs for (A) overall survival in patients with a LRR, (B) overall survival in patients with a SP, (C) survival after recurrence in patients with a LRR, and (D) survival after recurrence for patients with a SP tumour. Abbreviations: DFI = disease-free interval, LRR = locoregional recurrence, SP = second primary.

Ten year overall survival was 84% for women without recurrence, 49% for women with a LRR and 72% for women with a SP tumour. Ten year overall survival for patients with a LRR and a short DFI was 31%, 50% for medium and 70% for women with long DFIs (Fig 1A). Patients with a SP tumour demonstrated higher ten year overall survival rates with 69% for short DFIs, 72% for medium and 76% for long DFIs (Fig 1B). Table 5 shows the multivariable Cox regression analyses. Compared to short DFIs, women with a medium DFI and a LRR showed better survival after recurrence (Fig 1C) (Hazard Ratio (HR) 0.81, 95% CI 0.65–1.01) as well as women with a long DFI (HR 0.65, 95% CI 0.48–0.88). No significant association was found for the DFI and survival after SP tumours after adding covariates (Fig 1D).

**Table 5:** Multivariable Cox regression analysis after multiple imputation.

		LRR (n = 890)						SP (n = 745)					
		Univariable			Mutivariable			Univariable			Multivariable		
		HR	95% CI	P	HR	95% CI	P	HR	95% CI	P	HR	95% CI	P
**DFI**													
	Short	Ref.			Ref.			Ref.			Ref.		
	Medium	0.63	0.51–0.78	<0.001	0.81	0.65–1.01	0.066	1.26	0.90–1.78	0.184	1.14	0.80–1.61	0.475
	Long	0.39	0.29–0.52	<0.001	0.65	0.48–0.88	0.005	1.37	0.93–2.01	0.113	1.42	0.96–2.09	0.079
**Type of recurrence**													
	LR	Ref.			Ref.			na			na		
	RR	1.44	1.17–1.78	<0.001	0.83	0.64–1.07	0.144						
	Synchronous LRRs	2.03	1.47–2.83	<0.001	1.99	1.41–2.79	<0.001						
**Age**													
	<70 years	Ref.			Ref.			Ref.			Ref.		
	≥70 years	2.28	1.86–2.79	<0.001	2.29*	1.85–2.84	<0.001	2.59*	1.93–3.48	<0.001	2.28*	1.67–3.12	0.002
**Grade**													
	I, II	Ref.			Ref.			Ref.			Ref.		
	III	2.61*	2.12–3.21	<0.001	1.42	1.10–1.82	0.006	1.45	1.05–2.00	0.023	1.12	0.75–1.67	0.570
**Tumour size**													
	≤2 cm	Ref.			Ref.			Ref.			Ref.		
	>2 cm	2.60	2.13–3.18	<0.001	1.47	1.19–1.83	<0.001	1.76	1.32–2.36	<0.001	1.42	1.03–1.96	0.033
**Lymph node status**													
	Negative	Ref.			Ref.			Ref.			Ref.		
	1–3 positive	1.71	1.37–2.14	<0.001	1.15	0.86–1.54	0.355	1.66	1.19–2.32	0.003	1.36	0.92–2.02	0.123
	>3 positive	4.06	3.17–5.20	<0.001	2.06	1.48–2.88	<0.001	1.89	1.17–3.06	0.010	1.05	0.61–1.82	0.864
**Hormone receptor status**													
	Other	Ref.			Ref.			Ref.			Ref.		
	ER/PR negative	2.49	2.02–3.06	<0.001	1.64	1.29–2.08	<0.001	1.45	0.99–2.13	0.055	1.30	0.82–2.05	0.268
**Lymph node dissection**													
	No	Ref.			Ref.			Ref.			Ref.		
	Yes	2.09	2.02–3.06	<0.001	1.40	1.05–1.86	0.023	1.80*	1.36–2.39	<0.001	1.28	0.90–1.83	0.170
**Radiotherapy of LRR/SP**													
	No	Ref.			Ref.			Ref.			Ref.		
	Yes	1.09	0.90–1.32	0.381	0.94	0.76–1.16	0.575	0.73	0.55–0.97	0.032	1.04	0.76–1.41	0.812
**Surgery of LRR/SP**													
	No	Ref.			Ref.			Ref.			Ref.		
	Yes	0.42*	0.35–0.51	<0.001	0.49*	0.39–0.61	<0.001	0.13*	0.09–0.19	<0.001	0.17*	0.11–0.27	<0.001

Abbreviations: HR = hazard rate, CI = Confidence Interval, DFI = disease-free interval, LR = local recurrence, RR = regional recurrence, LRR = locoregional recurrence, SP = second primary, ER = oestrogen receptor, PR = progesterone receptor, na = not applicable.

* indicates most influencing factors.

Convergence of the imputed dataset was adequate and the results of the Cox proportional hazards model on the multiple imputed data were comparable to those of the complete case analysis. Continuous DFI (expressed in days) also displayed significant influence in the multivariable analysis for LRR (HR 0.9997 per day), but did not result in a better model fit. There was no interaction identified for the complete model. The Cox proportional hazards assumption was not violated when tested both numerically and graphically. The variables that influenced survival after recurrence the most were age category and whether or not the LRR or SP tumour had been surgically removed.

## Discussion

To the best of our knowledge, this is the first time the relation between the DFI and survival after a LRR or SP tumour has been explored on a large scale in a nationwide population-based registry. The DFI before a LRR is a prognostic factor for overall survival and survival after a LRR, with longer DFIs showing longer survival. Besides the DFI, age and surgical removal of the recurrence were significantly associated with survival after recurrence. After adding covariables in the multivariable analysis, length of the DFI before a LRR remained of significant influence on survival after the LRR, which suggests that it is an independent prognostic factor. No significant independent association was found for the DFI and survival after SP tumours. Patients with a short DFI and a LRR show on average less differentiated cells (grade III) and a larger primary tumour compared to the patients with medium and long DFIs, which suggests a more biological aggressive form of breast cancer. Moreover, short DFIs are significantly associated with subsequent DM [16, 25]. Another possible explanation is that patients with a short DFI still had to recover fully from the treatment given for the primary tumour. In the multivariable analysis, the effect of the DFI on survival after a SP tumour was not significant. SP tumours are new breast cancers independent of the primary tumour and the prognosis is therefore also independent of the DFI after the primary tumour.

Consistent with previous studies, age <70, good differentiation of the tumour cells (grade I,II), smaller tumour size, negative lymph nodes, positive hormone status, no axillary lymph node dissection, radiotherapy and surgical removal of the recurrence have been identified to be associated with better survival [16–18, 26]. Both chemotherapy and hormone therapy are known to have a positive influence on survival after primary breast cancer [19, 27]. Yet after adding covariates in the proportional hazards models chemotherapy and hormone therapy were not of significant influence on survival after a LRR or a SP tumour. In the univariable analysis women who received chemotherapy or hormone therapy showed worse survival compared to those without (HR 1.61, 95% CI 1.37–1.90 for chemotherapy; HR 1.45, 95% CI 1.23–1.73 for hormone therapy). Recurrence of the breast cancer, possibly even during the adjuvant treatment period, might suggest resistance to the treatment [28, 29]. The SP tumours were in 95% of the cases contralateral. Most of the patients with a SP tumour (96%) had undergone surgery, which is associated with higher survival after the SP tumour. However, this positive effect cannot solely be contributed to the influence of the surgery, but also the selection of patients that are eligible for surgery. Of the patients with a LRR, 41% did not receive surgery for their recurrence. In case of a short DFI this proportion was 49%. However, this could be explained by the higher amount of non-breast conserving surgeries for the primary tumour in this group (68% received mastectomy). Typical reasons for withholding from surgical treatment include diffuse infiltration, inflammatory changes, subsequent DM, high age or patient preference [30, 31]. Besides less surgery, women with a short DFI also received less hormone therapy for their LRR (Table 2). Consistent with previous studies we found that women with hormone negative primary tumours have on average a shorter DFI, which resulted in less hormone therapy in this group [32]. The cohort represented patients who were diagnosed in the period 2003–2006 and treated at that time, which could explain the lower percentage of hormone and chemotherapy compared to current treatment (data not shown). Another reason is the exclusion of patients who received neo-adjuvant systemic therapy (4% of the patients), while it is uncertain with which stage the patients should be included in the model. LRRs and SP tumours show differences in characteristics and prognosis and therefore need a different approach. In some studies the distinction between SP tumours and LRR is not made [14, 16, 33], which might overestimate the prognosis after a LRR.

The advantage of using the large cohort from the population-based NCR—including almost all breast cancer patients diagnosed between 2003 and 2006—is the reflection of daily practice. However, only limited information was available regarding the characteristics of the recurrences. The median overall survival in this study is 8.1 years, which could be considered short in a tumour with such a high ten year survival rate. Still, the balance between sufficiently long follow-up and relevance of the results for the present clinical situation needs to be considered. The changes and improvements in the treatment of breast cancer in more recent years can, by definition, not be reflected in a study with a long follow-up. Since only first or synchronous recurrences are registered by the NCR, no correction is possible for subsequent recurrences. DM after breast cancer is considered responsible for most of the morbidity and virtually all mortality associated with breast cancer. If a LRR was followed by a DM, this would drastically influence prognosis [34, 35]. Therefore, an improvement of the registry would be to register not only first but also possible subsequent recurrences. Furthermore, knowledge about cause-specific death can help improve future research. Because of the observational design and a lack of detailed information on breast cancer-specific survival and comorbidity, results should be interpreted with caution. However, Doyle et al. [36] found no difference in cause-specific and overall survival after a recurrence in the first five years and only a 3% difference after ten years.

## Conclusion

This is the first study to explore the association between the DFI and survival after recurrence in a nationwide population-based registry and it contributes to insight into prognosis after LRR or SP breast cancer. The results of this study confirm that the DFI before a LRR is an independent prognostic factor for survival after a LRR, with longer DFIs showing longer survival. Furthermore, age and surgical removal of the recurrence greatly impact survival after recurrence. However, no significant independent association was found for the DFI and survival after SP tumours.
